# An Approach for Easy Detection of Buried FRP Composite/Non-Metallic Pipes Using Ground-Penetrating Radar

**DOI:** 10.3390/s23208465

**Published:** 2023-10-14

**Authors:** Jonas Kavi, Udaya B. Halabe

**Affiliations:** 1 Civil & Environmental Consultants, Inc., Bridgeport, WV 26330, USA; jkavi@cecinc.com; 2 School of Sustainable Engineering and the Built Environment, Ira A. Fulton Schools of Engineering, Arizona State University, Tempe, AZ 85287, USA

**Keywords:** ground-penetrating radar (GPR), corrosion, excavation damage, fiber-reinforced polymer (FRP) composites, carbon-fiber-reinforced polymer (CFRP), glass-fiber-reinforced polymer (GFRP), polyvinyl chloride (PVC), pipelines

## Abstract

Pipelines remain the safest means of transporting natural gas and petroleum products. Nonetheless, the pipeline infrastructure in the US is facing major challenges, especially in terms of corrosion of steel/metallic pipes and excavation damage of onshore pipelines (leading to oil spills, explosions, and deaths). Corrosion of metallic pipelines can be avoided by using non-corrosive materials such as plastic pipes for low-pressure applications and glass-fiber-reinforced polymer (GFRP) composite pipes for transporting high-pressure oil and natural gas. However, buried non-metallic pipelines are not easily detectable, which can lead to increased excavation damage during construction and rehabilitation work. Alternative strategies for making buried non-metallic pipes easily locatable using ground-penetrating radar (GPR) were investigated in this study. Results from this study have shown that using carbon fabric or an aluminum foil overlay on non-metallic pipes before burying in soil significantly increases the reflected GPR signal amplitude, thereby making it easier to locate such pipelines. The reflected GPR signal amplitude for pipe sections with carbon fabric or aluminum foil overlays was found to have increased by a factor of up to 4.5 over the control samples. The results also highlight the importance of selecting the appropriate antenna frequency for GPR surveys, since wet silt loam soil and clay significantly reduce the penetration depths of the radar signals produced by the GPR antennae.

## 1. Introduction

Corrosion of metallic pipelines and excavation damage of onshore pipelines are among the major challenges that threaten the safe and expedient operation of transportation pipelines [1]. Steps are being considered to incorporate non-metallic materials into the development and construction/rehabilitation of pipelines to avoid threats posed by corrosion [2,3]. Thus, significant efforts are being made toward the development and/or use of fiber-reinforced polymer (FRP) composite pipes for high-pressure applications and plastic pipes for low-pressure applications [2,3,4]. Glass- and carbon-fiber-reinforced polymer (GFRP and CFRP) composite pipes offer several advantages such as high specific strength, high specific modulus, low specific weight, high corrosion resistance, high fatigue strength, and low coefficient of thermal expansion compared to traditional materials like steel [5,6]. Many research studies have shown the advantages of using composites for civil infrastructure applications [7,8,9,10,11,12,13,14]. Research has shown that CFRP cable with a bending anchorage system has a good fatigue-bearing capacity [7]. Another study investigated the durability of GFRP bars and carbon/glass-hybrid-fiber-reinforced polymer bars. This research study has shown that “CFRP layer enhances the durability of GFRP bars in alkaline environment” [8], which is “due to the delayed water and OH– diffusion in CFRP and the better alkaline resistance of CFRP” [8]. Further studies conducted on the residual strength and bond properties of GFRP bars have shown that “vinyl ester GFRP bars retain a significant percentage of their tensile strength and elastic modulus after they have cooled from elevated temperatures up to 400 °C” [9]. Another study investigated the effect of elevated temperatures on composite elastic properties [10]. This study showed a negligible change in the elastic modulus of CFRP plates, while the tensile strength and elongation of the same specimen only showed a gradual decrease with change in temperature from 10 °C to 90 °C [10]. Glass and carbon fabrics are also used in FRP composites to wrap concrete structural members to repair and/or strengthen them [11,12,13,14].

GFRP has a competitive cost advantage over CFRP; thus, GFRP is preferred for most infrastructure applications even though its strength is less than that of CFRP [2]. While non-metallic pipes such as glass-fiber-reinforced polymer and plastics are resistant to corrosion degradations in the field, they pose a challenge for subsurface detection and mapping since these materials are not easily detectable using the available ground sensory technologies [1,15]. This can lead to increased excavation damage during construction and rehabilitation work. It should be noted that the placement of metallic tracer wires over non-metallic pipes (buried underground) does not provide a viable long-term solution for pipe detection, since the metallic tracer wires corrode and break after some time, while the buried pipelines are expected to be in service for over 50 years.

This research focuses on the use of ground-penetrating radar (GPR) for the location of buried non-metallic pipes. GPR is a very versatile technique that has been successfully employed in many infrastructure condition assessment and geophysical survey applications [16,17,18,19,20,21,22,23,24,25,26,27,28,29,30,31,32,33,34,35,36,37,38]. These applications include pavement thickness measurement [16], defect detection in concrete or composite bridge decks [17,25], reinforcing steel location in concrete [22,23,34], archaeological investigation [18,19], detection of landmines [28], snow thickness measurement and/or subglacial topography detailing [27,31], tree root detection [20,21,26], irrigation and soil water content monitoring [36,37], detection of cracks in concrete or pipe walls [24,30], and underground/concrete void detection [29,38]. GPR has also been used for buried container and pipe/utility detection [15,32,33,35], but these applications include the detection of metal pipes or pipes filled with water, which are much easier to detect using GPR compared to non-metallic buried pipes, which may not have water inside it.

This paper investigates alternative strategies for creating easily locatable glass-fiber-reinforced polymer (GFRP) composite pipes and Polyvinyl Chloride (PVC) pipes that could help address the corrosion and excavation damage problems related to the use of metallic pipes. This experimental study involved the development, investigation, and comparison of different methods for locating buried non-metallic pipes through GPR surveys. These methods included the use of carbon fabric and aluminum foil overlays on non-metallic pipes before burying them. Aluminum foil is a cheap material that can be used as overlay on PVC pipes. On the other hand, CFRP fabric is more expensive, but it is a durable material; hence, it is preferred for use with GFRP pipes. It is important to note that a pipe made entirely out of CFRP composite (an electrical conductor) would be detectable using GPR, but the cost of CFRP pipe is much higher compared to GFRP pipe with some bonded CFRP material.

## 2. Transmission and Reflection of GPR Signals

**Figure 1:**
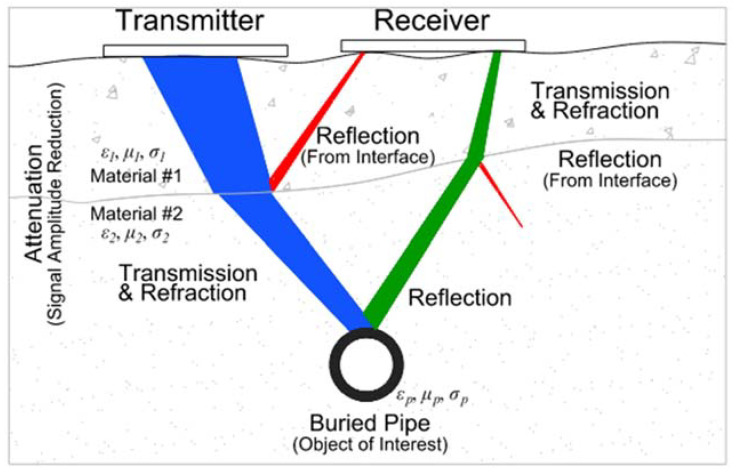
Propagation path of electromagnetic wave from transmitter to receiver.

The ground-penetrating radar technique operates by transmitting electromagnetic pulses from an antenna into a material and measuring the reflected signal response from subsurface features/interfaces. The reflected signal amplitude, phase, and elapsed time of the received signal are all recorded by the receiving antenna. The recording of successive reflections from different depths into a material produces a signal trace, which helps in locating subsurface features and objects. The amplitude of the reflected signal depends on the reflection coefficient and the incident signal amplitude at the interface. In the case of subsurface objects/features, the incident signal amplitude at an interface is controlled by the reflection, transmission, and attenuation properties of the overlaying material (Figure 1). The reflection coefficient directly correlates with the difference/contrast between the electromagnetic wave impedance of the two media at an interface. For an electromagnetic wave travelling from medium 1 to medium 2, the reflection coefficient, *R*_1,2_, for the signal amplitude reflected by the interface between the two media can be expressed as [36,39]

(1) $$R_{1,2}=\frac{Z_{2}-Z_{1}}{Z_{2}+Z_{1}}\;$$

where *Z*_1_ and *Z*_2_ are the electromagnetic wave impedances of the first and second medium at the interface, respectively.

Generally, *Z* for a medium is defined as

(2) $$Z=\sqrt{\frac{\mu_{m}^{\prime }}{\varepsilon_{m}^{\prime }+i\frac{\sigma^{\prime }}{\omega }}}$$

where:

$\mu_{m}^{\prime }$ is the absolute magnetic permeability of the medium (H/m);
$\varepsilon_{m}^{\prime }$ is the absolute dielectric constant/permittivity (F/m);
*σ’* is the electrical conductivity (S/m);
*ω* is the angular frequency of the radar wave (rad/s);
*i* is the imaginary unit ($=\sqrt{-1}$).

The relative (dimensionless) electromagnetic properties of a medium ($\mu_{r}^{\prime }\;\mathrm{and}\;\varepsilon_{r}^{\prime }$) are defined as

(3) $$\mu_{r}^{\prime }=\frac{\mu_{m}^{\prime }}{\mu_{o}}\;\;\Rightarrow \;\mu_{m}^{\prime }=\mu_{r}^{\prime }\mu_{o}\;\;$$

(4) $$\varepsilon_{r}^{\prime }=\frac{\varepsilon_{m}^{\prime }}{\varepsilon_{o}}\;\;\Rightarrow \;\;\varepsilon_{m}^{\prime }=\varepsilon_{r}^{\prime }\varepsilon_{o}\;$$

where:

$\mu_{r}^{\prime }$ is the relative magnetic permeability of the medium;
$\varepsilon_{r}^{\prime }$ is the relative dielectric permittivity of the medium (also referred to as dielectric constant);
*μ_o_* = 4π × 10^−7^ H/m is the magnetic permeability of vacuum or free space;
*ε_o_* = 8.854 × 10^−12^ F/m is the dielectric permittivity of vacuum or free space.

For a low-loss dielectric material with low electrical conductivity (*σ*′), Equation (2) becomes

(5) $$Z=\sqrt{\frac{\mu_{m}^{\prime }}{\varepsilon_{m}^{\prime }}}$$

The magnetic properties of most soil are negligible; thus, $\mu_{m}^{\prime }$ = *μ_o_* = 4π × 10^−7^ H/m and $\mu_{r}^{\prime }$ = 1 [36]. It can be shown from the above discussion that the reflection coefficient for media with low conductivity and low magnetic permeability can be simplified as [39,40]

(6) $$R_{1,2}≅\frac{\sqrt{\varepsilon_{1}^{\prime }}-\sqrt{\varepsilon_{2}^{\prime }}}{\sqrt{\varepsilon_{1}^{\prime }}+\sqrt{\varepsilon_{2}^{\prime }}}$$

The transmission coefficient at the interface is given by [39]

(7) $$T_{1,2}=\frac{2Z_{2}}{Z_{2}+Z_{1}}≅\frac{2\sqrt{\varepsilon_{1}^{\prime }}}{\sqrt{\varepsilon_{1}^{\prime }}+\sqrt{\varepsilon_{2}^{\prime }}}$$

where subscripts 1 and 2 denote the first and second medium at the interface, respectively.

From Equations (1) and (6), it can be seen that a higher difference between the electromagnetic wave impedances of the two media at an interface would result in a higher reflection coefficient; hence, a greater percentage of the incident wave amplitude would be reflected back to the receiving antenna. This study investigates different approaches for increasing the electromagnetic wave impedance difference or contrast at the interface between buried pipes and the surrounding soil.

## 3. Experimental Program

The detectability of buried PVC, GFRP, and CFRP pipes with different external surface overlays/finishes was investigated using GPR under different soil moisture conditions. A total of thirty-nine pipe samples of different diameters were prepared and buried at various depths in the field. Material properties, sample preparations, and the experimental setup for this study are discussed next.

### 3.1. Pipe Material Preparation

Pipe samples used in this research had diameters of 30.48 cm, 15.24 cm, and 7.62 cm (12″, 6″, and 3″, respectively) and were made from PVC, GFRP, and CFRP materials. A steel pipe of 30.48 cm diameter (12″) and a GFRP pipe of 25.40 cm (10″) diameter were also buried to serve as control specimens. All the pipe samples were 152.4 cm (5 ft) long and capped at both ends to prevent ground water from filling them when buried since the objective of this study was to locate the pipes without relying on any benefit that may be provided by the pipe content. Preparation of the various pipe samples for GPR testing is elaborated below.

The 30.48 cm and 15.24 cm (12″ and 6″, respectively) diameter PVC pipe samples (152.4 cm or 5 ft long) were cut from pipes with the SDR-35 specification, while the 7.62 cm (3″) diameter PVC pipes were obtained from schedule 40 (SCH 40) pipes. These PVC pipes were procured from a local store that supplies common construction materials.

The 30.48 cm (12″) and 25.40 cm (10″) diameter GFRP pipes, as well as the 7.62 cm (3″) diameter CFRP pipes, were supplied by a composites product manufacturer, while the 30.48 cm (12″) diameter CFRP pipe and 7.62 cm (3″) diameter GFRP pipe were produced by wrapping two layers of carbon fabric (for CFRP pipe) and two layers of glass fabric (for GFRP pipe) with resin around molds and then curing them to obtain the required pipes. Material properties of the fabrics and resin (matrix) system used in producing the composite pipes are shown in Table 1.

### 3.2. Creating Dielectric Contrast between Non-Metallic Pipes and Surrounding Soil

Non-metallic pipe materials (such as PVC and GFRP) buried underground are generally not detectable using GPR in most soil conditions. This is because PVC and GFRP pipe materials are non-conductive, have similar electromagnetic impedance as most soils, and are generally transparent to radar waves and hence do not reflect the incident waves under most soil conditions. In order to make these pipe materials detectable using GPR in their buried state, the strategy would be to create or increase the contrast between the electromagnetic wave impedances of the pipes and the surrounding soil (simply referred to as dielectric contrast hereafter). Three different approaches were adopted to create dielectric contrast between the pipe materials and the sounding soil—using CFRP rings or strips, aluminum rings or strips, and carbon nanoparticle overlays.

Carbon fiber, aluminum, and carbon nanoparticles, like steel, are electrical conductors and as such do not allow the significant transmission of radio waves such as GPR signals through them. GPR signals incident on these conductive materials produce a strong reflection signal that is received by a receiver, thereby making the material detectable in their underground state. This behavior is different from the surrounding soil, which absorbs and/or allows the signal to travel through it.

**Figure 2:**
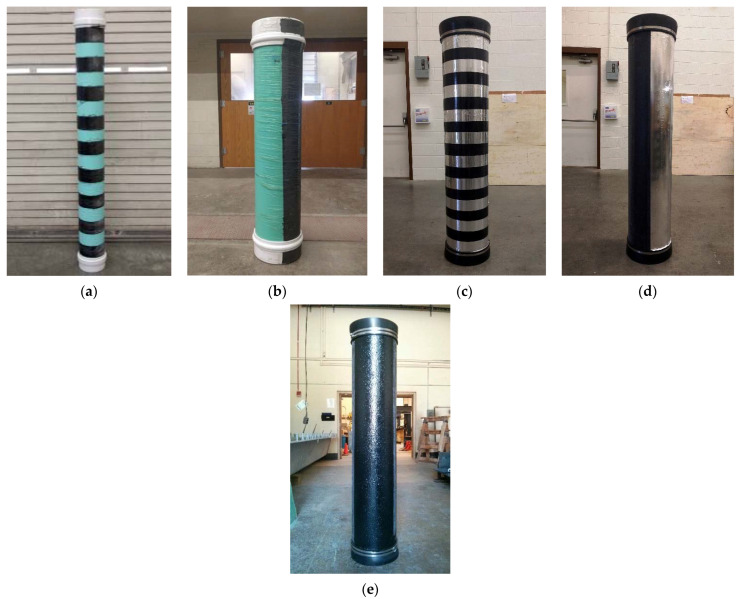
Pipe configurations: (**a**) 15.24 cm (6″) diameter polyvinyl chloride (PVC) pipe with carbon-fiber-reinforced polymer (CFRP) rings; (**b**) 30.48 cm (12″) diameter PVC pipe with CFRP strip; (**c**) 30.48 cm (12″) diameter glass-fiber-reinforced polymer (GFRP) pipe with aluminum rings; (**d**) 30.48 cm (12″) diameter GFRP pipe with aluminum strip; and (**e**) 30.48 cm (12″) diameter GFRP pipe with carbon nanoparticle overlay.

The dielectric contrast between the PVC and GFRP pipes, and the surrounding soil, was created by wrapping the pipes with carbon fabric in two different configurations. The first configuration involved wrapping 7.62 cm (3″) wide carbon fabric around three of the PVC pipe samples (one each of 30.48 cm, 15.24 cm, and 7.62 cm diameters) and two of the GFRP pipe samples (30.48 cm and 7.62 cm diameters) in the form of rings, at a clear spacing of 7.62 cm (3″). The second configuration involved bonding a carbon fabric strip at the top of the pipes along the full lengths (excluding pipe caps in most cases) of three PVC and two GFRP pipe samples. For the 30.48 cm (12″) diameter pipes, the widths of the strips were up to half the circumference of the pipes. The width of the CFRP strips were up to ¾ of the circumference of the 15.24 cm (6″) diameter pipes, and the strips covered the entire circumference in the case of the 7.62 cm (3″) diameter pipes. This was performed to ensure that part of the CFRP strip remains at the top of the pipe after burying even if the pipe rotates during backfilling. Figure 2a,b show the 15.24 cm (6″) diameter PVC pipe with the 7.62 cm (3″) wide CFRP rings and the 30.48 diameter (12″) PVC pipe with the CFRP strip, respectively. Material properties of the fabrics and resin system used in creating the CFRP strips and rings are shown in Table 1.

Similar to the carbon fabric, aluminum foil was wrapped around some of the PVC and GFRP pipes (in the form of strips and rings) to create a dielectric contrast between the pipes and the surrounding soil when buried. The first configuration involved wrapping 7.19 cm (2.83″) wide aluminum foil tapes around three PVC pipe samples (30.48 cm, 15.24 cm, and 7.62 cm diameters) and two GFRP pipe samples (30.48 cm and 7.62 cm diameters), at a clear spacing of 7.62 cm (3″). The second configuration involved placing an aluminum foil strip along the lengths of three PVC pipes and two GFRP pipe samples similar to the carbon fabric strips. Figure 2c,d show a 30.48 cm (12″) diameter GFRP pipe with aluminum rings and a 30.48 cm (12″) diameter GFRP pipe with an aluminum strip along the length, respectively. The aluminum foil overlays will be especially useful for PVC pipes since these aluminum foil tapes are readily available in hardware stores that sell PVC pipes, and the aluminum tapes can be bonded easily to the already manufactured PVC pipes.

Carbon nanoparticles are also expected to be electrical conductors; thus, it is anticipated that the interconnection between individual carbon nanoparticles in a coating/overlay will be able to reflect the incident GPR signal and make the buried non-metallic material with this coating detectable as a result. To test the effectiveness of this approach, a 30.48 cm (12″) diameter GFRP pipe was coated with a mixture of carbon nanoparticles and vinyl ester resin, up to a quarter of the pipe circumference and through the entire length of the pipe (excluding end caps), as shown in Figure 2e. The objective here was to investigate if this configuration created an adequate dielectric contrast between the pipe and the surrounding soil when buried.

**Figure 3:**
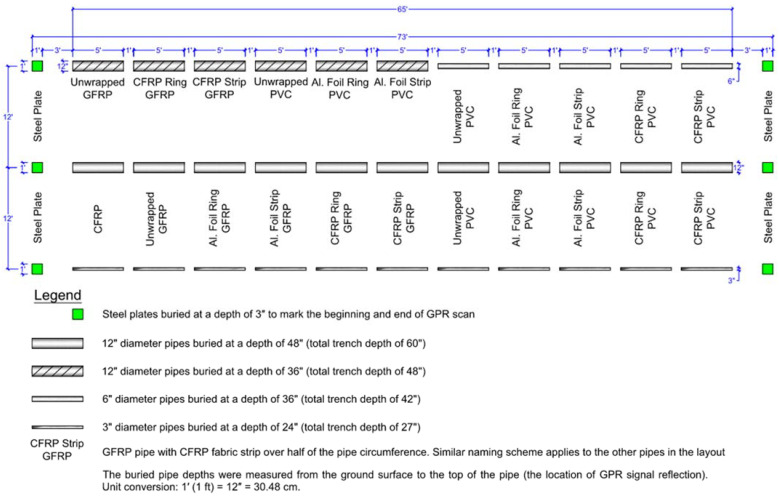
Pipe layout for GPR testing (three long trenches).

The surface wraps or overlays (CFRP and aluminum strips or rings, and carbon nanoparticle coating) are electrically conductive, and are therefore expected to reflect incident radar waves much better than from the surface of buried non-metallic pipes without such overlays. Better reflection of the radar waves from these conductive overlays will make it much easier to locate the buried non-metallic pipes using GPR. Some of the PVC and GFRP pipes were not wrapped (these are labeled as “Unwrapped” in Figure 3) and were used as control specimens during GPR detectability testing of the samples.

### 3.3. Pipe Setup and Burying

**Figure 4:**
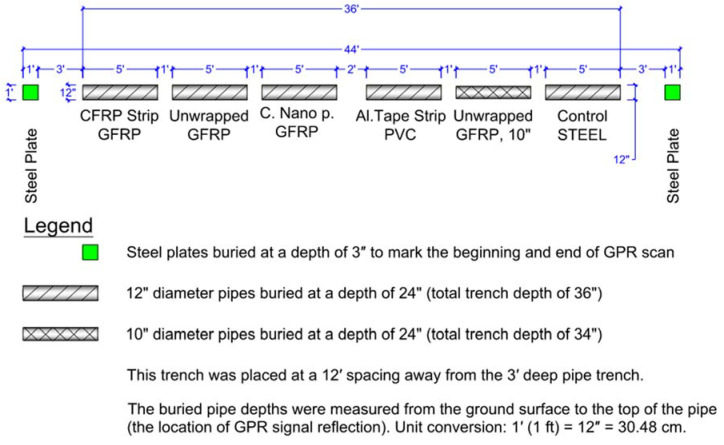
Pipe layout for GPR testing (one short trench).

The ends of all the pipes were capped before burying so that the pipes only had air inside them, and water would not go inside the pipes in the buried state. This was performed to ensure that GPR would detect the pipe material without relying on the pipe’s interior content (such as water or any other liquid). The pipe segments were buried in three separate 19.81 m (65 ft) long trenches and one short trench of 10.97 m (36 ft) length. The four trenches were spaced at 3.66 m (12 ft) apart center-to-center. Each of the first three trenches (19.81 m long) contained 11 pipe segments, with a 30.48 cm (1 ft) spacing between each subsequent pipe, as shown in Figure 3. The fourth trench (10.97 m long) had six buried pipe segments, with a 30.48 cm (1 ft) spacing between subsequent pipes and a 60.96 cm (2 ft) spacing between pipes at the middle of the trench, as shown in Figure 4. Some of the 30.48 cm (1 ft) diameter and 152.4 cm (5 ft) long PVC, CFRP, and GFRP pipes were buried at a depth of 121.92 cm (4 ft) in the second trench (total trench depth of 152.4 cm or 5 ft), as shown in Figure 3. In all cases, the buried pipe depth was measured from the ground surface to the top of the pipe, which is the location of GPR signal reflection from the pipe. Two different-diameter pipes, 30.48 cm (12″) and 15.24 cm (6″), were buried in the first trench (Figure 3), with both buried at a depth of 91.44 cm (3 ft) to the top of the pipe. The third trench had 7.62 cm (3″) diameter pipes buried at a depth of 60.96 cm or 2 ft (total trench depth of 68.58 cm or 27″), as shown in Figure 3. The fourth trench (10.97 m long trench) contained 30.48 cm (1 ft) diameter GFRP, PVC, steel, and 25.4 cm diameter GFRP pipes buried with 60.96 cm (2 ft) of soil cover depth. The complete layout of the pipe samples, including the pipe material, diameter, pipe surface configuration, and depth of soil cover over buried pipes, is shown in Figure 3 and Figure 4. A summary of the pipe samples and configurations is also provided in Table 2.

**Figure 5:**
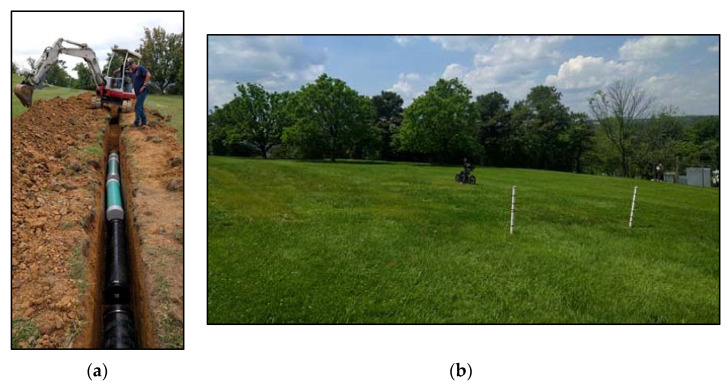
Pictures showing a trench and the backfilled test site ready for GPR testing. (**a**) Picture of a trench showing placement of pipes. (**b**) Site after soil backfilling and grass seeding.

Figure 5a shows a picture of one of the trenches with pipe placement. In addition to burying the pipes, two 30.48 cm (1 ft) wide steel plates were buried at 7.62 cm (3″) depth to mark the beginning and end of each trench (one steel plate at each end of the trench) for GPR testing, as shown in Figure 3 and Figure 4. After placing the pipes at their respective depths, the trenches were backfilled, compacted, leveled, and seeded with grass to restore the field to its original condition, as shown in Figure 5b. Leveling the ground and seeding with grass were performed to ensure that the site mirrors actual field conditions for buried pipes that need to be detected for the pipeline industry using GPR. The presence or absence of grass (bare area with dirt) would not have any significant effect on GPR detectability (as long as the grass is mowed and there is no tall grass), but in this case, the grass seeding was performed to match the conditions of the entire field (so that there is no variability in soil moisture content, due to absence of grass over the trenches). The results obtained from GPR testing will give an indication of the applicability of the developed techniques in the pipeline industry. GPR testing of buried pipes commenced after the grass had grown over the trenches and the entire field looked uniform in terms of its surface appearance.

This study’s test site consisted of organic soil characterized as Zoar silt loam [41] with a thickness of about 0.3 m (1 ft) at the top followed by clay up to 1.22 m (4 ft), which was the maximum depth considered in this study. Sites containing clay pose great difficulty for GPR investigation since clay attenuates the radar signals considerably and limits the penetration depth. On the other hand, sandy soil allows deeper penetration using GPR. In real life, buried pipes are located in a variety of soil conditions ranging from sandy soil to clayey soil. This paper investigates the difficult case of detecting buried pipes in clayey soil overlaid with silt loam.

It is important to note that radar wave propagation in soil is affected by the soil’s dielectric constant and electrical conductivity. Soil properties such as bearing strength do not affect radar wave propagation characteristics. Section 4.1 provides details of the soil dielectric properties at the test site used in this study.

### 3.4. GPR Test Equipment and Data Acquisition Parameters

A versatile piece of GPR equipment (manufactured by Geophysical Survey Systems, Inc., Nashua, NH, USA) with a changeable antenna setup, and capable of operating with different antenna frequencies, was used in this research for evaluating the appropriate choice of antenna for detection of buried non-metallic pipes. A 200 MHz radar antenna with the manufacturer’s specified penetration depth of up to 9 m and a 400 MHz antenna with the manufacturer’s specified penetration depth of up to 4 m were evaluated for pipe detection [42]. In addition to these antenna frequencies, a 900 MHz antenna with the manufacturer’s specified penetration depth of up to 1 m was also evaluated [42]. The maximum penetration depths specified by the manufacturer are typically for dry sandy soil. The actual penetration depth depends on the complex dielectric permittivity of the soil medium, and therefore can be significantly lower in soils with high moisture and/or high clay contents such as in the test site used in this study, as described in Section 3.3.

**Figure 6:**
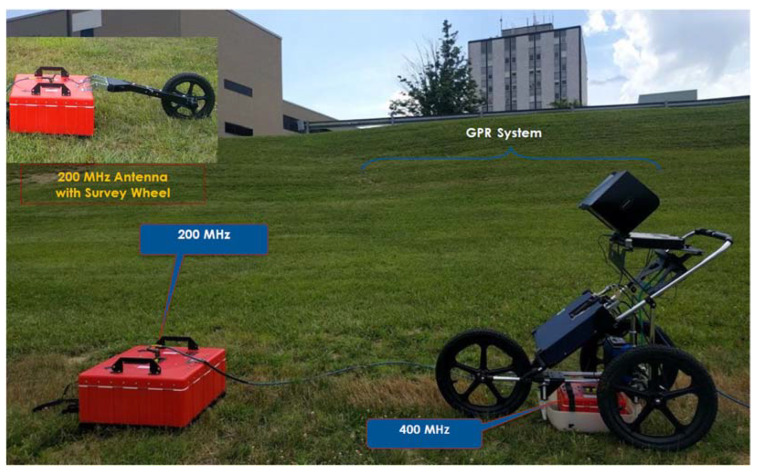
GPR test equipment shown on the test site with buried pipes.

In this study, each GPR test utilized the one-antenna mode, that is, the same antenna served as the transmitter and receiver (normal incidence). The GPR system had survey wheels with optical encoders for tracking the horizontal distance along the ground surface. A survey wheel attached to the GPR cart was used to track the horizontal distance when the 400 MHz or 900 MHz antenna was used, while the 200 MHz antenna had a survey wheel attached to the antenna itself for horizontal distance measurement (Figure 6). The GPR data acquisition parameters used in this study for the various antennas are shown in Table 3.

**Table 3:** GPR data acquisition parameters.

Parameters	900 MHz	400 MHz	200 MHz
Vertical Low-Pass Filter (MHz)	2500	800	400
Vertical High-Pass Filter (MHz)	225	100	50
Range (ns)	30	30 or 50	50
Samples per Scan	512	512	512
Bits per sample	16	16	16
Dielectric Constant	Varies, see Table 4	Varies, see Table 4	Varies, see Table 4
Scans/in.	12	12	12
Scans per Second	100	100	100
Transmit Rate (kHz)	100	100	100

**Table 4:** Average soil dielectric properties during GPR data collection.

Depth Up to	Dataset I			Dataset II			Dataset III		
cm (ft)	VWC, *θ* (m^3^/m^3^)	Diel. $\varepsilon_{r}^{\prime }$	Cond. *σ*′ (mS/m)	VWC, *θ* (m^3^/m^3^)	Diel. $\varepsilon_{r}^{\prime }$	Cond. *σ*′ (mS/m)	VWC, *θ* (m^3^/m^3^)	Diel. $\varepsilon_{r}^{\prime }$	Cond. *σ*′(mS/m)
61 (2)	0.290	13.42	10.94	0.473	26.77	17.08	0.343	16.60	12.47
91 (3)	0.315	15.09	11.43	0.473	26.75	16.97	0.363	18.12	12.86
122 (4)	0.341	16.76	11.92	0.473	26.73	16.85	0.383	19.65	13.25

### 3.5. Soil Moisture and Electrical Conductivity Sensor

**Figure 7:**
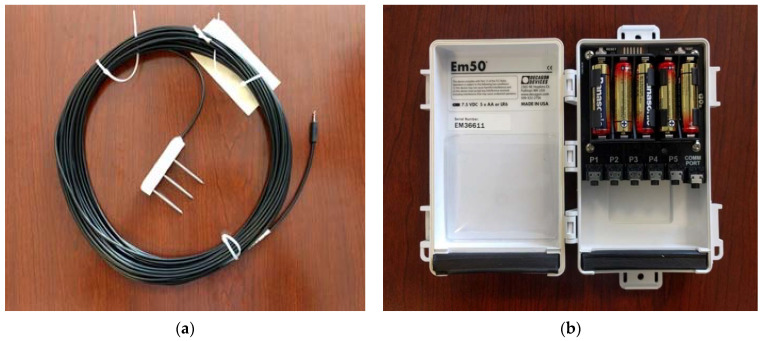
Sensor for measuring dielectric constant, electrical conductivity, temperature, and volumetric water content of soil. (**a**) Soil moisture and dielectric permittivity sensor. (**b**) Data logger.

The ground-penetrating radar’s signal attenuation, penetration depth, and amplitude of received signals are affected by the dielectric properties of the soil medium under investigation. Thus, knowledge of the soil properties such as dielectric constant and electrical conductivity are essential for the effective planning and execution of successful GPR surveys. In this study, five soil sensors were placed in the trenches at different depths to measure soil properties throughout the testing period. Two of the sensors were buried at a 122 cm (4 ft) depth near the 30.48 cm (1 ft) diameter pipes, and two more sensors were buried at a 61 cm (2 ft) depth near the 7.6 cm (3″) diameter pipes. In addition, one soil sensor was used to measure soil properties at various locations on the ground surface. Figure 7a shows the soil sensor and Figure 7b shows the five-channel data logger, with the sixth channel (communication port) used to connect the data logger to a laptop computer for data acquisition. The soil sensors enabled quantitative determination of the volumetric water content, electrical conductivity, temperature, and relative dielectric permittivity (also known as dielectric constant) of the soil during the testing period. Knowledge of the soil parameters enabled accurate estimation of the depth of soil cover over the pipes using GPR. These soil parameters were also used in numerical computations of radar signal attenuation and skin depths during data analysis.

## 4. Results and Analysis

### 4.1. Soil Dielectric Properties and GPR Experiments

GPR surveys were conducted over buried pipes under different soil moisture conditions (indicated by the changing soil volumetric water content, which led to different dielectric properties for each test, as provided in Table 4) and using different antennae frequencies (200 MHz and 400 MHz). A 900 MHz antenna was also evaluated at the beginning of this study and found to be inadequate in achieving the expected penetration depth in the relatively wet soil medium in this study’s test site, which consisted of silt loam (about 0.3 m or 1 ft deep) at the top followed by clay up to 122 cm (4 ft), which was the maximum depth considered in this study. GPR scans were carried out in both the longitudinal direction along the pipe trenches and transverse direction across the trenches/pipes.

The average volumetric water content (VWC or *θ*), dielectric constant (*ε_r_*′), and electrical conductivity (*σ*′) of the soil up to different depths measured during the GPR survey using the soil sensors (Figure 7) are provided in Table 4. The VWC significantly affects the dielectric constant and electrical conductivity of the soil medium. The soil properties for 61 cm (2 ft) depth are the averages of soil sensor readings at the surface (0 ft) and at 61 cm (2 ft) depth. This would allow depth determination from GPR signals for pipes buried at 61 cm (2 ft) depth. Similarly, the soil properties listed in Table 4 for 122 cm (4 ft) depth are averages of all the measurements in the 0–122 cm (0–4 ft) range and used for pipes buried at 122 cm (4 ft) depth. The soil properties for the 0–91 cm (0–3 ft) range were obtained by taking average of the properties for 0–61 cm (0–2 ft) and 0–122 cm (0–4 ft) ranges.

Datasets I and III represent relatively dry conditions, while Dataset II represents the wet condition of the soil. Some of the test data collected during the GPR survey in relatively dry soil in the summer months (Dataset I) are presented and discussed in this paper. Results from GPR surveys conducted in relatively wet soil in the winter months (Dataset II) and in the spring months (Dataset III) were found to be consistent with Dataset I in terms of depth predictions [1].

### 4.2. Results from GPR Experiments Using 200 MHz Antenna

**Figure 8:**
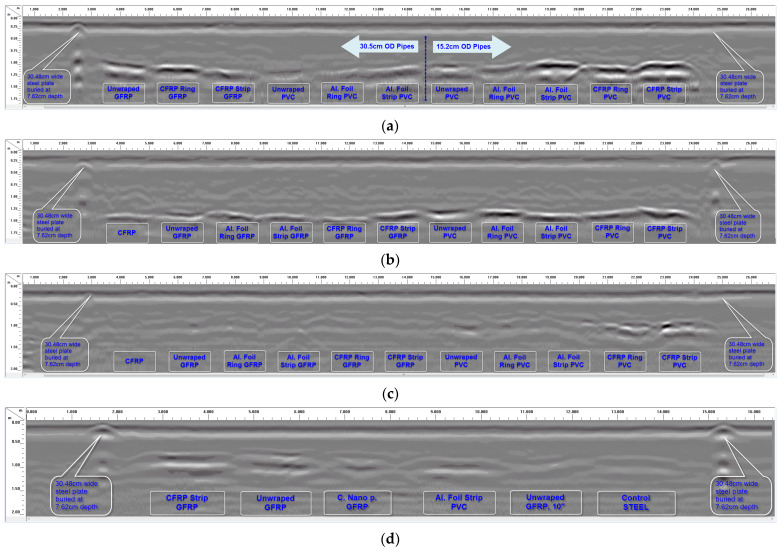
Longitudinal GPR scans over the pipe trenches using 200 MHz antenna. (**a**) Longitudinal GPR scan along the full length of 30.48 cm (12′) and 15.24 cm (6″) diameter pipes with 91.44 cm (3 ft) of soil cover. (**b**) Longitudinal GPR scan along the full length of 30.48 cm (12″) diameter pipes with 122 cm (4 ft) of soil cover. (**c**) Longitudinal GPR scan along the full length of 7.62 cm (3″) diameter pipes with 61 cm (2 ft) of soil cover. (**d**) Longitudinal GPR scan along the full length of 30.48 cm (12″) and 25.4 cm (10″) diameter pipes with 61 cm (2 ft) of soil cover.

**Figure 9:**
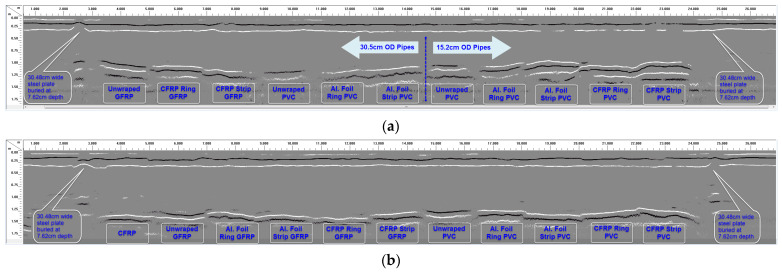

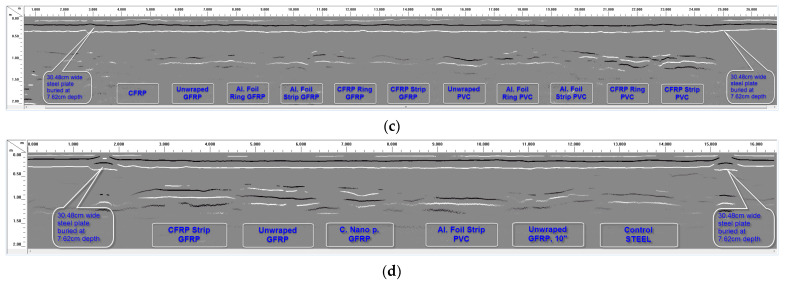
Reflected signal peaks extracted from GPR scans in Figure 8. (**a**) Peak extraction processing used to make buried pipes in Figure 8a more visible. (**b**) Peak extraction processing used to make buried pipes in Figure 8b more visible. (**c**) Peak extraction processing used to make buried pipes in Figure 8c more visible. (**d**) Peak extraction processing used to make buried pipes in Figure 8d more visible.

Raw data from longitudinal GPR scans over the four trenches using the 200 MHz antenna are shown in Figure 8. In Figure 8 (and the rest of the GPR data), “CFRP Ring GFRP” means GFRP pipe wrapped with CFRP/carbon fabric rings; “CFRP Strip GFRP” means GFRP pipe bonded with a CFRP fabric strip at the top; and “Unwrapped PVC” means PVC pipe without any surface wrap (control sample). Similar naming schemes are used for all the other pipes in the figure. Figure 9 shows the scans from each trench processed using the “peaks extraction” data processing technique (a specified number of reflected signal peaks were extracted along the depth while the remaining low-amplitude peaks were set to zero). The objective of conducting the longitudinal scans was to compare the radar reflections from different pipe overlay configurations along the same line. This helped in understanding which overlay configurations produced stronger GPR reflections.

With the exception of the 7.62 cm (3″) diameter pipes buried with 61 cm (2 ft) of soil cover over the pipes (Figure 8c), good GPR signal reflections were recorded from pipes buried in all the other trenches, as shown in Figure 8a,b,d. These reflected signals made it possible to detect the buried pipes with varying levels of clarity and signal strengths. It is also observed from Figure 8 and Figure 9 that carbon fabric and aluminum foil wraps/overlays on the pipe sections improve detectability with GPR. It is further observed that carbon fabric strips and aluminum strips along the full length of the pipe sections generally produce better results compared to aluminum rings and carbon fabric rings; this aspect is explained further in the following paragraphs. GPR data from the 30.48 cm (12″) and 15.24 cm (6″) diameter pipes buried with 91.44 cm (3′) of soil cover, and 30.48 cm (12″) and 25.4 cm (10″) diameter pipes buried with 61 cm (2′) of soil cover are evaluated next. More details on the test data can be found in a publication by [1].

As shown in Figure 8a and Figure 9a, all the buried pipes with 91.44 cm (3′) of soil cover (with the exception of 30.48 cm (12″) diameter PVC pipe without any overlay and 30.48 cm diameter PVC pipe with aluminum foil rings) can be detected with varying levels of clarity in the raw GPR data when scanned with the 200 MHz radar antenna. Particularly, pipe sections with CFRP and aluminum foil overlays (strips or rings) show up prominently with higher signal strengths in the GPR scan. These pipes are easily detected compared to pipes without any overlay. Among the pipes with CFRP or aluminum foil overlays, CFRP and aluminum foil strips along the full length of the pipes are generally easier to detect compared to CFRP and aluminum foil rings around the pipes. Post-processing the data using peak extraction can make it easier to locate the buried pipes, as shown in Figure 9a. Peak extraction also makes it possible to locate pipes that were otherwise not visible in the original (raw) GPR scan (compare Figure 9c to Figure 8c).

**Figure 10:**
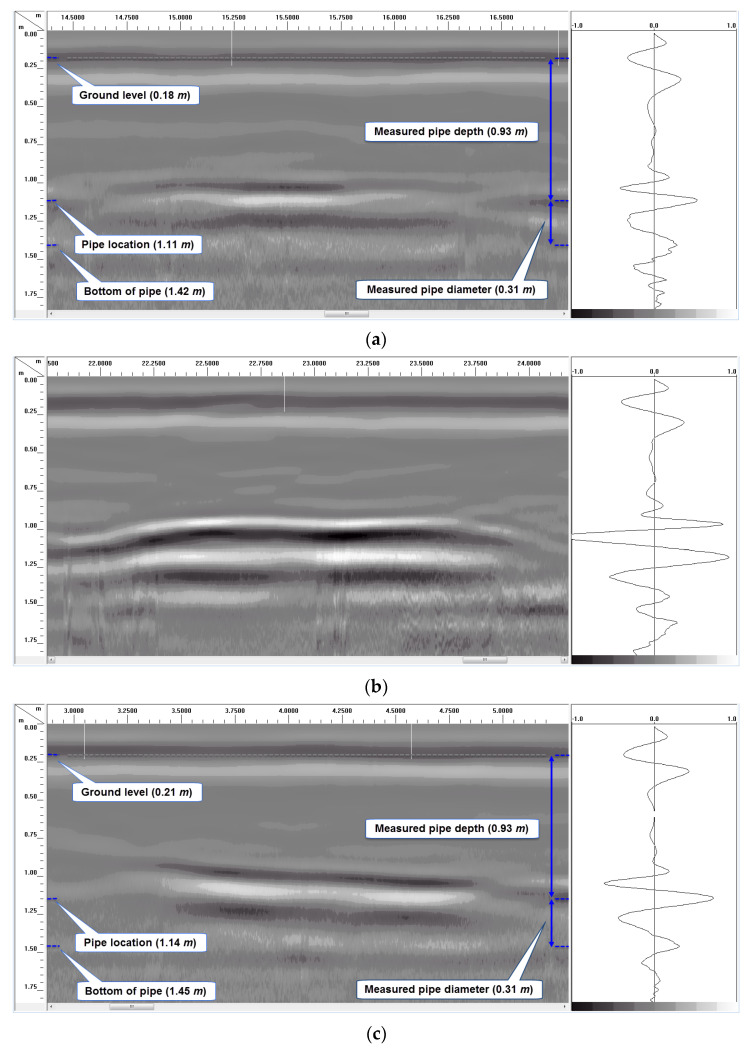

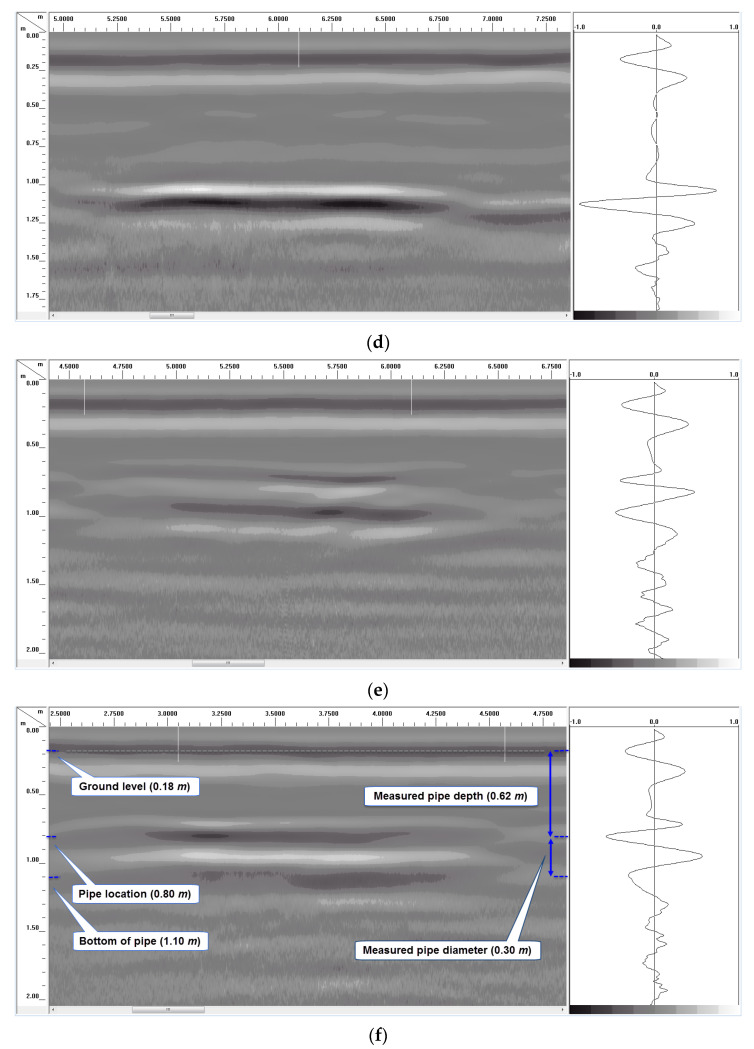
Detailed longitudinal GPR B-Scan (left) and A-Scan (right) over some of the buried pipes using 200 MHz antenna. (**a**) Longitudinal GPR scan over 15.24 cm (6″) unwrapped PVC pipe with 91.44 cm (3 ft) of soil cover. (**b**) Longitudinal GPR scan over 15.24 cm (6″) CFRP strip PVC pipe with 91.44 cm (3 ft) of soil cover. (**c**) Longitudinal GPR scan over unwrapped 30.48 cm (12″) GFRP pipe with 91.44 cm (3 ft) of soil cover. (**d**) Longitudinal GPR scan over 30.48 cm (12″) CFRP ring GFRP pipe with 91.44 cm (3 ft) of soil cover. (**e**) Longitudinal GPR scan over 30.48 cm (12″) unwrapped GFRP pipe with 61 cm (2 ft) of soil cover. (**f**) Longitudinal GPR scan over 30.48 cm (12″) CFRP strip GFRP pipe with 61 cm (2 ft) of soil cover.

Figure 10a–d show details of some of the pipe scans in Figure 8a, including the B-Scan to the left and A-Scan (individual GPR signal over the center of the pipe) to the right of each figure. By using the measured soil dielectric constant (Table 4) and round-trip travel time for normal incidence (one antenna method) obtained from the A-Scans, the depth of soil cover over the pipes was accurately estimated, as shown in Figure 10a,c,f, where the measured pipe depths of 0.93 m and 0.62 m using the GPR signal correlates very well with the actual pipe depths of 0.91 m (3′) and 0.61 m (2′), respectively. In this study, direct measurement of the soil dielectric constant using buried soil sensors during GPR surveys enabled the pipe depths (soil cover over the top of the pipes) to be estimated. Other methods such as hyperbolic fitting, the common midpoint method (CMP), and Wide-Angle Reflection and Refraction (WARR) can be used to estimate the pipe depths from GPR data in the absence of accurate dielectric constant measurements or estimates [43,44]. GPR reflections from both the top and bottom of some of the pipes make it possible to estimate the diameters of such pipes, as shown in Figure 10a,c,f, where the measured pipe diameters of 0.31 m and 0.30 m correlate very well with the actual pipe diameter of 0.3048 m (12″). However, since the diameter of the pipe is estimated from the electromagnetic wave velocity, which is based on the average soil dielectric constant in this case, there is the possibility for the estimated diameter to be less accurate. For more precise estimation of the pipe diameter in the field, the velocity of the material inside the pipe should be used instead of the soil velocity. Also, it is important to note that reflected signals from the top and bottom of the pipe could overlap for smaller-diameter pipes (15 cm or less) with low-frequency (~200 MHz) GPR signals, which could affect travel time determination through the diameter of the pipe.

The unwrapped 15.24 cm (6″) diameter PVC produced a very weak signal reflection in the GPR scan at 91.44 cm of depth (Figure 10a). Although the weak signal makes it possible to detect the pipe in this scan, bonding it with a CFRP strip along the length (at the top) makes it a lot easier to locate the pipe, as shown in Figure 10b. This pipe with a CFRP strip at the top shows up prominently in the GPR scan and is detected with a clean reflected signal from the top of the pipe. Strong reflection from the top of the pipe will make it possible to locate the pipe with GPR irrespective of the content of the pipe.

Figure 10c,d show detailed GPR results from 30.48 cm (12″) diameter GFRP pipes with and without CFRP rings. Similar to the previous comparison, the pipe with the CFRP rings around it produced a GPR reflection with a much higher amplitude (Figure 10d) compared to the pipe without any carbon fabric rings (Figure 10c). Also, it is interesting to note that the soil–CFRP boundary results in peak reversal (phase reversal of reflected signal’s peak from positive to negative) for the reflection from the pipe, as seen by comparing the A-scans (signal to the right) in unwrapped and wrapped cases (Figure 9d and Figure 10c). Such a peak reversal can also be seen by comparing Figure 10a with Figure 10b.

As shown in Figure 8d and Figure 9d, all the 30.48 cm (12″) and 25.4 cm (10″) diameter pipes with 61 cm (2 ft) of soil cover are detectable in the data because of the shallow burial depth, but with varying levels of clarity when scanned with the 200 MHz radar antenna. Similar to the previous discussions, the GFRP pipe with the CFRP strip at the top and the PVC pipe with the aluminum foil strip at the top are prominently visible. The returned signal from the 30.48 cm (12″) diameter GFRP pipe with no external wrap is also very good; however, the reflection from top of the pipe is short, or not continuous over the entire length of the pipe (only signal reflection from the bottom of the pipe is continuous through the full length of the pipe). The GFRP pipe with the carbon nanoparticle overlay produced a good but very short reflection from the top of the pipe, as shown in Figure 8d. It was concluded that the carbon nanoparticles mixed with resin did not possess a significant level of electrical conductivity, due to a lack of connectivity between the nanoparticles, unlike the CFRP fabric with continuous carbon fibers. Thus, it was concluded that the effectiveness of the carbon nanoparticle coating is low in terms of producing strong GPR signal reflections. The unwrapped 25.4 cm (10″) diameter GFRP pipe and the steel pipe produced weak reflections, with the steel pipe being a bit more visible in the GPR scans shown in Figure 8d and Figure 9d.

Figure 10e,f show GPR scans of two of the 30.48 cm (12″) diameter pipes buried with 61 cm (2 ft) of soil cover (pipe with and without CFRP strip); both pipes can be detected in the GPR scans. The pipe without the CFRP strip at the top produced a short and weaker signal corresponding to the top of the pipe (see the B-Scan in Figure 10e) compared to the stronger and continuous reflection from the pipe bottom. Though the pipe is detected using the combination of top and bottom reflections in this case, it will be difficult to locate the pipe if its content absorbs the radar signal and makes it impossible to obtain reflections from the bottom of the pipe. On the contrary, the GFRP pipe with the CFRP strip at the top is detected with a strong and continuous reflected signal from the top and bottom of the pipe, as seen in the B-Scan in Figure 10f. Prominent reflection from the top of the pipe will make it possible to locate the pipe with GPR irrespective of the content of the pipe.

**Figure 11:**
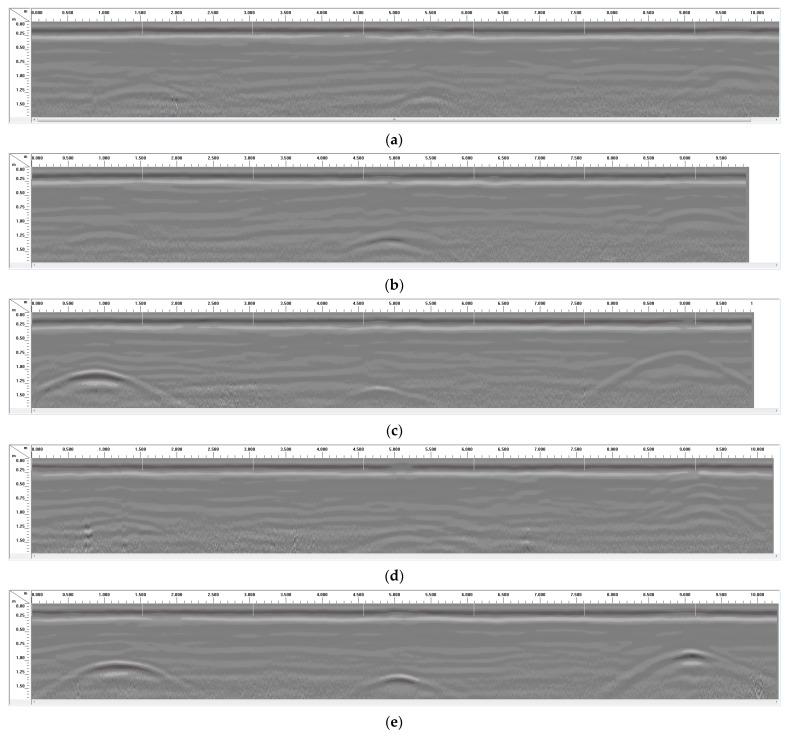
Transverse GPR scans over the pipes in 19.81 m (65 ft) long trenches using 200 MHz antenna. (**a**) The 15.24 cm dia. unwrapped PVC at 91.44 cm, 30.48 cm dia. unwrapped PVC at 122 cm, and 7.62 cm dia. unwrapped PVC at 61 cm. (**b**) The 15.24 cm dia. Al foil ring PVC at 91.44 cm, 30.48 cm dia. Al foil ring PVC at 122 cm, and 7.62 cm dia. Al foil ring PVC at 61 cm. (**c**) The 15.24 cm dia. Al foil strip PVC at 91.44 cm, 30.48 cm dia. Al foil strip PVC at 122 cm, and 7.62 cm dia. Al foil strip PVC at 61 cm. (**d**) The 15.24 cm dia. CFRP ring PVC at 91.44 cm, 30.48 cm dia. CFRP ring PVC at 122 cm, and 7.62 cm dia. CFRP ring PVC at 61 cm. (**e**) The 15.24 cm dia. CFRP strip PVC at 91.44 cm, 30.48 cm dia. CFRP strip PVC at 122 cm, and 7.62 cm dia. CFRP strip PVC at 61 cm.

For most GPR applications in locating buried pipes or utility lines, scans are often performed perpendicular to the expected direction of the utility line (transverse scans). Thus, for Dataset III (see soil properties listed in Table 4), scans were also performed perpendicular to the direction of the pipes (transverse scans) for comparison. Figure 11a–e shows transverse GPR scans over some of the buried pipes in the first three trenches (19.81 m or 65 ft long trenches) with the pipe layout shown in Figure 3. Each of the transverse GPR scans (shown in Figure 11) was performed over all the three trenches, starting from the 91.44 cm (3 ft) deep trench and ending over the 61 cm (2 ft) deep trench (or from the top line of pipes to the bottom line of pipes according to the layout given in Figure 3). The 122 cm (4 ft) deep trench was in the middle of the three trenches. The five GPR transverse scans shown in Figure 11a–e correspond to the seventh pipe through the eleventh pipe (pipes on the right side of the layout in Figure 3).

Each of the five transverse GPR scans in Figure 11a–e correspond to three PVC pipes (15.24 cm, 30.48 cm, and 7.62 cm diameters from left to right) buried at different depths. The GPR scan in Figure 11a corresponds to unwrapped PVC pipes, while the other four GPR scans (Figure 11b–e) correspond to PVC pipes with different overlays such as aluminum foil or CFRP fabric in the form of rings around the pipe or strips over the pipe. Among all the GPR scans in Figure 11a–e, the weakest scans are observed in Figure 11a, which corresponds to the unwrapped PVC pipes.

Contrary to the unwrapped PVC pipes, the PVC pipes with aluminum foil or CFRP strips (Figure 11c,e) produced the strongest radar reflections among all the scans in Figure 11. The PVC pipes with CFRP strips also produced reflections with higher amplitudes at the apex of the hyperbola (Figure 11e) compared to the PVC pipes with aluminum foil strips (Figure 11c). Radar reflections from the PVC pipes with aluminum foil rings produced mixed results (Figure 11b), with only one of the pipes (middle pipe) showing a higher-amplitude signal compared to that obtained from the unwrapped PVC (Figure 11a). The GPR scan in Figure 11d shows that CFRP rings were not very useful in enhancing the GPR reflection of PVC pipes in a transverse scan. On the other hand, CFRP strips were very useful in producing high-amplitude reflections, as shown in the GPR scans in Figure 11e. Unlike the rings placed at discrete intervals, the strips provide a continuous reflector, which is crucial in the case of transverse GPR scanning.

### 4.3. Results from GPR Experiments Using 400 MHz Antenna

**Figure 12:**
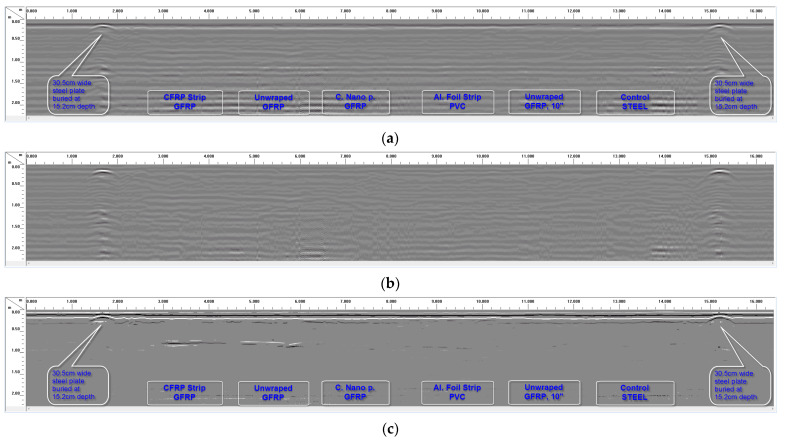
Longitudinal GPR scan over the 61 cm (2 ft) deep trench for Dataset I using 400 MHz antenna. (**a**) Longitudinal scan along the full length of 30.48 cm (12″) and 25.4 cm (10″) diameter pipes with 61 cm (2 ft) of soil cover. (**b**) Background noise removal applied to the scan in (**a**). (**c**) Peak extraction processing used to make buried pipes in scan (**a**) visible.

Figure 12a shows the raw GPR scan (obtained using 400 MHz antenna for soil Dataset I) from the 30.48 cm (12″) and 25.40 cm (10″) diameter pipes buried with 61 cm (2 ft) of soil cover, while Figure 12b,c show the data in Figure 12a processed using background noise removal and peak extraction, respectively. It is difficult to identify any of the pipes in Figure 12a,b, but extracting reflection peaks (Figure 12c) makes it possible to see the GFRP pipe wrapped with the CFRP strip and the GFRP pipe with no external surface wrap (unwrapped GFRP pipe). The PVC pipe with the aluminum foil strip is very faintly visible in the processed data in Figure 12c. The 400 MHz antenna could not locate any of the pipes buried beyond 61 cm (2 ft) of depth. Thus, the 400 MHz antenna was much less effective in locating the buried pipes at 61 cm (2 ft) of depth or deeper when scanned in the longitudinal direction along the pipes. Conducting scans in the transverse direction across the pipes using the 400 MHz antenna made it possible to detect the buried pipes at 61 cm (2 ft) of depth, though with much less clarity compared to results from the 200 MHz antenna (more details are available in [1]). This is because the 400 MHz radar signals have a significantly higher signal attenuation compared to the 200 MHz radar signals. Thus, a much greater portion of the transmitted signal energy is lost in the soil in the case of the 400 MHz antenna. The signal attenuation and performance of the different antennae are explained further in a later section of this paper.

### 4.4. Discussion on Performance of Surface Configurations

From the discussion of GPR test results presented above, the performance of the various pipe surface configurations investigated in this study can be summarized as follows:

1. Carbon fabric and aluminum foil overlays improved the detectability of buried non-metallic pipes using GPR.
2. Carbon fabric and aluminum foil strips along the full length of the pipe performed better than carbon fabric and aluminum foil rings at a regular spacing of 3″ (7.62 cm) around the pipes.
3. Carbon fabric overlays on pipes generally perform better than aluminum foil overlays.

The performance of carbon fabric and aluminum foil in improving the detectability of the buried non-metallic pipes can be attributed to the fact that carbon fabric and aluminum are good electrical conductors; hence, they reflect the incident radar waves significantly better than the non-conducting GFRP or PVC pipe material buried in soil. These higher-amplitude reflections from the conductive overlays are recorded by the receiving antenna, hence making it possible to locate the buried pipes.

**Figure 13:**
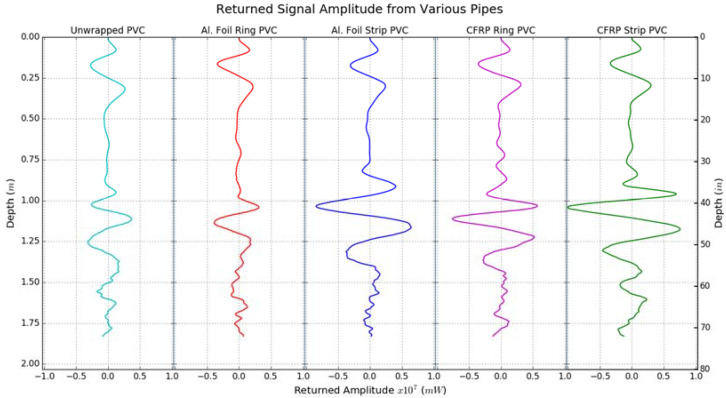
Comparison of received radar signal amplitude from different pipe configurations buried at 91.44 cm (3 ft) depth.

Figure 13 provides a comparison of the received radar signal amplitude from five different 15.24 cm (6″) diameter PVC pipes buried with 91.44 cm (3 ft) of soil cover. This plot corresponds to results from the 200 MHz GPR antenna for the soil conditions in Dataset I (see Table 4). This plot clearly shows the superior performance of carbon fabric and aluminum foil overlays in increasing the reflected signal amplitudes (compared to unwrapped PVC), thereby making buried pipes easier to detect during GPR surveys. Figure 13 also shows that the strips produced a stronger reflection compared to rings.

The performance of strips versus rings can be evaluated in two parts and explained by the following observations. For GPR scans conducted along the length of the pipe (longitudinal scans), a significant portion of the antenna’s electromagnetic beam fell on the long strips (about 1.37 m or 4.5 ft long strips, excluding pipe caps at the ends) and was reflected, as opposed to the 7.62 cm (3″) wide rings, which only covered a small portion of the antenna beam. For scans conducted perpendicular to the pipe direction (transverse scans), the rings only produced good signal reflections when the radar antenna was centered over a ring, as opposed to being centered between two rings. Hence, it is recommended that continuous conductive strip overlays be used instead of discrete rings to make non-metallic pipes detectable using GPR.

The addition of carbon fabric or aluminum foil overlays on buried pipes was found to increase the reflected signal amplitude by up to 4.5 times, and 2 times on average across all the pipe sections tested. Carbon fabric overlays performed better in terms of detectability with GPR compared to aluminum foil overlays because the carbon fabrics used were thicker than the aluminum foil. Increasing the thickness of the aluminum overlay for the pipes can improve their detectability when buried, as well as increase the durability of the aluminum overlay. However, this approach may not be practically feasible. Another important consideration is the durability of the overlay in the buried environment. Since metallic tracer wires have been found to be eroded in soil after a few decades, it is important to make sure that the overlay material is durable. Carbon fabric is a highly durable material and can last for many decades in buried conditions. While CFRP pipes can be used to replace metal pipes, it would be much more cost-effective to use GFRP pipes with the CFRP fabric overlay at the top to aid in GPR detection.

### 4.5. Signal Attenuation Modeling and Antenna Performance

GPR signals experience higher signal attenuation with increasing antenna frequency. Thus, lower-frequency antennae penetrate deeper than higher-frequency ones. The total signal attenuation consists of multiple components, including the ohmic (material) and scattering attenuations. Ohmic or material attenuation (Equation (8)) was found in this study to be constant for all antenna frequencies above 50 MHz and up to 2500 MHz considered for modeling, while scattering attention increased with antenna frequency [1]. Scattering attenuation due to the presence of air pockets and gravels in the soil were computed using “PyMieScatt” [45] version 1.7.1, which provides an implementation of Equations (9)–(15) [46,47,48] in an open source Python Mie Scattering module.

The material properties provided in Table 5 were used in addition to the soil dielectric properties provided in Table 4 (values for 0 to 122 cm depth range were used) for computing scattering attenuations due to the presence of gravels and air pockets in the soil. The dielectric constant of gravel shown in Table 5 is the average of the properties for gravel listed in the published literature [40,49,50,51].

(8) $$k_{I}=\omega \sqrt{\frac{\varepsilon_{m}^{\prime }\mu_{m}^{\prime }}{2}\left(\sqrt{1+{\left(\frac{\sigma^{\prime }}{\omega \varepsilon_{m}^{\prime }}\right)}^{2}{}_{\;}}-1\right)}\;\;$$

where:

*k_I_* is the ohmic (material) attenuation coefficient (m^−1^);
*ω* = 2πf is the angular frequency (rad/s);
*f* is the frequency of the electromagnetic wave (Hz);
$\varepsilon_{m}^{\prime }$ is the absolute dielectric permittivity of the soil (F/m);
$\mu_{m}^{\prime }$ is the absolute magnetic permeability of the soil (H/m);
*σ*′ is the electrical conductivity of the soil (S/m).

(9) $$k_{sca}=\frac{NC_{sca}}{2}\;$$

where *k_sca_* is the scattering attenuation (m^−1^), *N* is the number of particles per unit volume, and $C_{sca}$ is the scattering cross-section of scatter particles.

(10) $$C_{sca}=Ca^{6}f^{4}=\frac{2\pi }{k^{2}}\overset{\infty }{\underset{n=1}{\sum }}\left(2n+1\right)\left({\left|a_{n}\right|}^{2}+{\left|b_{n}\right|}^{2}\right)\;$$

(11) $$=\pi a^{2}\frac{2}{x^{2}}\overset{\infty }{\underset{n=1}{\sum }}\left(2n+1\right)\left({\left|a_{n}\right|}^{2}+{\left|b_{n}\right|}^{2}\right)$$

(12) $$x=ka=\frac{2\pi Na}{\lambda_{v}}=\frac{2\pi a}{\lambda }\;$$

(13) $$a_{n}=\frac{\mu m^{2}j_{n}\left(mx\right){\left[xj_{n}\left(x\right)\right]}^{\prime }-\mu_{1}j_{n}\left(x\right){\left[mxj_{n}\left(mx\right)\right]}^{\prime }}{\mu m^{2}j_{n}\left(mx\right){\left[xh_{n}^{\left(1\right)}\left(x\right)\right]}^{\prime }-\mu_{1}h_{n}^{\left(1\right)}\left(x\right){\left[mxj_{n}\left(mx\right)\right]}^{\prime }}$$

(14) $$b_{n}=\frac{\mu_{1}j_{n}\left(mx\right){\left[xj_{n}\left(x\right)\right]}^{\prime }-\mu j_{n}\left(x\right){\left[mxj_{n}\left(mx\right)\right]}^{\prime }}{\mu_{1}j_{n}\left(mx\right){\left[xh_{n}^{\left(1\right)}\left(x\right)\right]}^{\prime }-\mu h_{n}^{\left(1\right)}\left(x\right){\left[mxj_{n}\left(mx\right)\right]}^{\prime }}\;$$

(15) $$m=\frac{k_{1}}{k}=\frac{N_{1}}{N}\;\;$$

where:

*C* is a constant;
*a* is the radius of the scattering particle (m);
*f* is the frequency of the electromagnetic wave (Hz);
*k* is the wave number (m^−1^);
*μ*, *μ*_1_ are the magnetic permeabilities of the soil medium and particle, respectively;
*N*, *N*_1_ are the refractive indices of the soil medium and particle, respectively;
*λv*, *λ* are the wavelengths of the electromagnetic wave in vacuum and in the soil medium, respectively (m).

Skin depth, which is the depth or distance that a plane wave has to travel for its amplitude to reduce to 1/e or 36.8% of its original value, is defined as the inverse of the total attenuation coefficient (*k_I_* + *k_sca_*) [52]. The skin depth (m) and total signal attenuation coefficient (m^−1^) are useful in estimating the penetration depth and the likely amplitude of any GPR signal reflections [49].

**Figure 14:**
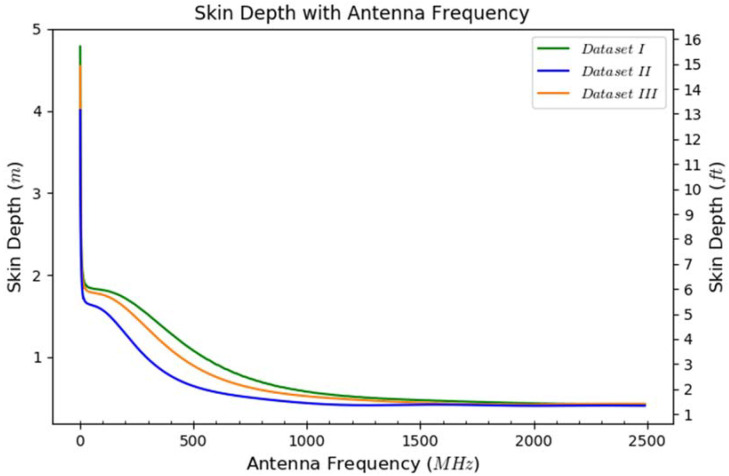
Variation in skin depth with antenna frequency for different soil volumetric water contents (see Table 4).

The variation in skin depth across antenna frequencies from 1 MHz to 2500 MHz for the three soil datasets (see Table 4) is provided in Figure 14. The skin depth decreases with increasing antenna frequency, as shown in Figure 14, and gives an indication of how each antenna performs with respect to depth. This also explains why the 400 MHz antenna was not able to locate any of the pipes buried deeper than 61 cm (2 ft), while the 200 MHz antenna offered deeper penetration in the soil. It should also be noted that the soil in Dataset II (which has the highest volumetric water content and the highest electrical conductivity, as shown in Table 4) results in the lowest skin depth, as seen in Figure 14.

It is also important to note that the signal amplitude reduces to 1/e or 36.8% of its original value at the skin depth, but in most radar applications (including this study), a round-trip travel of the radar signal is involved. This means that the received signal with round-trip travel to and from the target buried at the skin depth will have a signal amplitude equal to 1/e^2^ or 13.5% of the original (transmitted) signal amplitude.

## 5. Conclusions

From the GPR test results discussed in this paper, it is evident that the use of CFRP and aluminum foil/tape overlays significantly improves the detectability of buried non-metallic pipes such as GFRP and PVC. In cases where the buried unwrapped GFRP and PVC pipes were detectable (albeit with faint and difficult-to-interpret signals), the addition of carbon or aluminum foil overlays significantly increased the amplitude of the reflected GPR signal and made it much easier to identify the pipes. CFRP and aluminum foil overlays performed significantly better in making the buried non-metallic pipes detectable because these overlays are electrical conductors; hence, they reflect the incident radar waves much better than the non-conducting pipe material and the surrounding soil. The CFRP fabric or aluminum foil overlays were found to increase the reflected signal amplitude by up to 4.5 times, and 2 times on average across all the pipe sections tested.

The production of stronger and easier-to-interpret signals from buried non-metallic pipes with carbon fabric or aluminum foil overlays also implies that the depth of pipe burial can be increased beyond the 122 cm (4 ft) maximum depth evaluated in this research, and one can still obtain adequate signal strength using GPR.

Carbon fabric overlays were observed to produce stronger radar signal reflections from buried pipes compared to aluminum foil overlays. It was also observed that carbon fabric and aluminum foil strips bonded to the top of the pipes generally produced better/stronger signals compared to carbon fabric and aluminum foil rings (at a spacing of 3″ or 7.62 cm) around the non-metallic pipe sections.

The addition of a carbon nanoparticle coating on a GFRP pipe did not provide any noticeable benefit in making the non-metallic pipe detectable using GPR. This can be attributed to the lack of interconnection between the individual nanoparticles; hence, the coating did not act as a continuous conductor as was expected.

Furthermore, it was found from the GPR testing (from some tests that were conducted in winter months) that some snow cover on the ground surface does not hinder the performance of GPR in detecting the buried pipes. This can be attributed to the fact that the dielectric constant and electrical conductivity of snow are very low; hence, the GPR signal travels through the snow cover without much attenuation, and at higher signal velocity compared to the underlying soil medium. This is true as long as the ground is not frozen. If the ground is frozen, then a sharp boundary between frozen and unfrozen soil could pose a major problem for GPR detection since the boundary will cause unwanted reflection and result in a loss of energy incident on the buried pipes.

Finally, it was observed that the 200 MHz GPR antenna performed significantly better in locating all the buried pipes compared to the 400 MHz and 900 MHz antennae. This is because the signal from the higher-frequency antennae attenuates significantly more with respect to travel distance compared to the lower-frequency antenna. The 400 MHz antenna performed well in locating the pipes buried with 61 cm (2 ft) of soil cover (consisting of silt loam and clay with high moisture content), especially when scanned in the transverse direction, which is often the mode/direction of scanning during utility locating surveys. Only the 200 MHz antenna could locate pipes at depths of 91.44 cm (3 ft) and 122 cm (4 ft).

Based on this study, it is recommended that non-metallic pipes be overlaid with a conductive layer such as aluminum or carbon fabric before burying so that the buried pipes are easily detectable using GPR technique. Also, the soil type and moisture content would significantly affect the penetration depth capability of GPR antennas.

## Figures and Tables

**Table 1:** Material and section properties of carbon-fiber-reinforced polymer (CFRP) and glass-fiber-reinforced polymer (GFRP) pipes/fabrics.

Pipe Section	Wall Thickness, mm (in.)	Fiber Material	Fiber Mat (Fiber Orientation)	Fiber Weight, g/m^2^ (oz/yd^2^)	Resin (Matrix) Material
**30.48 cm (12″) GFRP**	9.525 (3/8)	E-Glass	45°/90°/−45°	^†^	Polyurethane
**25.40 cm (10″) GFRP**	9.525 (3/8)	E-Glass	Filament wound	^†^	Vinyl Ester
**7.62 cm (3″) GFRP**	**	E-Glass	0°/90°	813.74 (24)	Vinyl Ester
**30.48 cm (12″) CFRP**	**	Carbon	0°/90°/±45°	949.36 (28)	Vinyl Ester
**CFRP Strip/Ring**	*	Carbon	0°/90°/±45°	949.36 (28)	Vinyl Ester
**7.62 cm (3″) CFRP**	7.938 (5/16)	Carbon	^†^	^†^	^†^

* One layer of fabric was used. ** Two layers of fabric were used. ^†^ Information was not provided by the composite pipe manufacturer.

**Table 2:** Summary of pipe samples and configurations.

		Pipe Diameters with Each Surface Configuration, cm						
Pipe Materials	Soil Cover Depth, m (ft)	Control (Unwrapped)	CFRP		Aluminum		Carbon Nano-Particle	Total
			Strip	Ring	Strip	Ring		
CFRP	1.22 (4)	30.48						1
	0.61 (2)	7.62						1
GFRP	1.22 (4)	30.48	30.48	30.48	30.48	30.48		5
	0.91 (3)	30.48	30.48	30.48				3
	0.61 (2)	30.48, 25.40, 7.32	30.48, 7.32	7.62	7.62	7.62	30.48	9
PVC	1.22 (4)	30.48	30.48	30.48	30.48	30.48		5
	0.91 (3)	30.48, 15.24	15.24	15.24	30.48, 15.24	30.48, 15.24		8
	0.61 (2)	7.62	7.62	7.62	30.48, 7.32	7.62		6
Steel	0.61 (2)	30.48						1
Total number of pipes		12	7	6	7	6	1	39

**Table 5:** Material properties for scattering attenuation computation.

Material Name	Dielectric $\mathrm{Constant},\;\varepsilon_{r}^{\prime }$	Conductivity, *σ*′ (mS/m)	Particle Diameter (mm)
Air Pockets	1.00	0.00	8
Gravels	5.25	0.0001	20
Soil Medium	From Table 4	From Table 4	NA

## Data Availability

The data presented in this study are available in the article.
